# Calibration of quartz tuning fork spring constants for non-contact atomic force microscopy: direct mechanical measurements and simulations

**DOI:** 10.3762/bjnano.5.59

**Published:** 2014-04-23

**Authors:** Jens Falter, Marvin Stiefermann, Gernot Langewisch, Philipp Schurig, Hendrik Hölscher, Harald Fuchs, André Schirmeisen

**Affiliations:** 1 Center for Nanotechnology (CeNTech) and Institute of Physics, University of Münster (WWU), Heisenbergstrasse 1, 48149 Münster, Germany; 2 Institute of Applied Physics (IAP), Justus-Liebig-University Gießen, Germany; 3 Institute of Microstructure Technology (IMT), Karlsruhe Institute of Technology (KIT), Hermann-von-Helmholtz Platz 1, 76344 Eggenstein-Leopoldshafen, Germany; 4 Institute of Nanotechnology (INT), Karlsruhe Institute of Technology (KIT), Hermann-von-Helmholtz Platz 1, 76344 Eggenstein-Leopoldshafen, Germany

**Keywords:** atomic force microscopy, calibration, instrumentation

## Abstract

Quartz tuning forks are being increasingly employed as sensors in non-contact atomic force microscopy especially in the “qPlus” design. In this study a new and easily applicable setup has been used to determine the static spring constant at several positions along the prong of the tuning fork. The results show a significant deviation from values calculated with the beam formula. In order to understand this discrepancy the complete sensor set-up has been digitally rebuilt and analyzed by using finite element method simulations. These simulations provide a detailed view of the strain/stress distribution inside the tuning fork. The simulations show quantitative agreement with the beam formula if the beam origin is shifted to the position of zero stress onset inside the tuning fork base and torsional effects are also included. We further found significant discrepancies between experimental calibration values and predictions from the shifted beam formula, which are related to a large variance in tip misalignment during the tuning fork assembling process.

## Introduction

Atomic force microscopy (AFM) allows the imaging of surfaces with true atomic resolution and the resolution of intra-molecular structures of molecules [1]. Furthermore, the non-contact AFM (nc-AFM) technique has the capability of quantifying the interaction forces acting between the probing tip and the sample site with atomic precision. Recent achievements of this force spectroscopy method manifest in the identification of the chemical identity of single atoms in an alloy [2] or the measurement of the force applied during the controlled manipulation of molecules or atoms on a surface [3–4]. nc-AFM experiments at the atomic scale usually demand well defined environments, such as ultrahigh vacuum (UHV) and low temperatures (LT). For these conditions, force sensors based on quartz tuning forks in the “qPlus” design [5] have been proven to routinely provide stable operation and sufficient sensitivity to achieve the highest resolution in nc-AFM experiments. Today, many commercially available AFMs for UHV and LT conditions are based on quartz sensors because of their impressive performance and easy technical implementation.

Common AFM sensors are microfabricated from silicon or silicon nitride with the tip already integrated. Their spread in geometric parameters is within a low range and the characterization of their geometric parameters has been presented extensively by theory and experiments [6–8]. Quartz tuning fork force sensors in contrast are usually hand-made and even though they are commercially available, they are far from mass production and therefore exhibit a large spread of geometric – and thus of elastic parameters. Especially the precise knowledge of the sensor stiffness *k*_qPlus_ is crucial for quantitative interpretation of force spectroscopy measurements. Early spectroscopy experiments compared relative forces with high accuracy, for which the absolute stiffness of the sensor was not critical. Latest measurements of the absolute interaction forces impress by their force resolution [3–4,9] but suffer from the large error and spread in the determination of the geometric factors of the “qPlus” sensors. The stiffness of the force sensor is necessary for the transformation of the experimental frequency shift data, Δ*f*, to forces. Consequently, a force measurement can only be as precise as the determination of each factor in the equation that links the frequency shift to the tip–sample forces [8,10–11]. To calculate the force-vs-distance curve from measured frequency shift-vs-distance data, the inversion of the dependence of the frequency shift on the tip–sample forces has been derived [11–14] with high accuracy. All those formulas contain the stiffness of the sensor *k*_qPlus_ as prefactor and therefore directly suffer from an inaccurate determination of the spring constant.

Here we present an experimental procedure that allows for the direct measurement of the stiffness of a tuning fork sensor in the “qPlus” design with standard lab equipment. Our results reveal that a large spread of stiffness exists even in a series of commercially sold sensors. This finding underpins the urge of the individual characterization of each sensor. The standard equation [15] to calculate the stiffness from the geometric dimensions is the beam formula. Comparison of our experimental results with the formula show large discrepancies up to a factor of 5. In the next step we use extensive finite element method (FEM) modeling of the precise geometry of the tuning fork sensor in order to understand these deviations. The simulations show quantitative agreement with the beam formula if the beam origin is shifted to the position of zero stress onset inside the tuning fork base and torsional effects are included as well. Comparison with experimental spring constant data still show that the spring constant is overestimated by FEM and beam formula. This effect is attributed to a small but not negligible angle between the tip wire axis and the surface normal of the tuning fork prong.

## Results and Discussion

### Experiment

**Figure 1:**
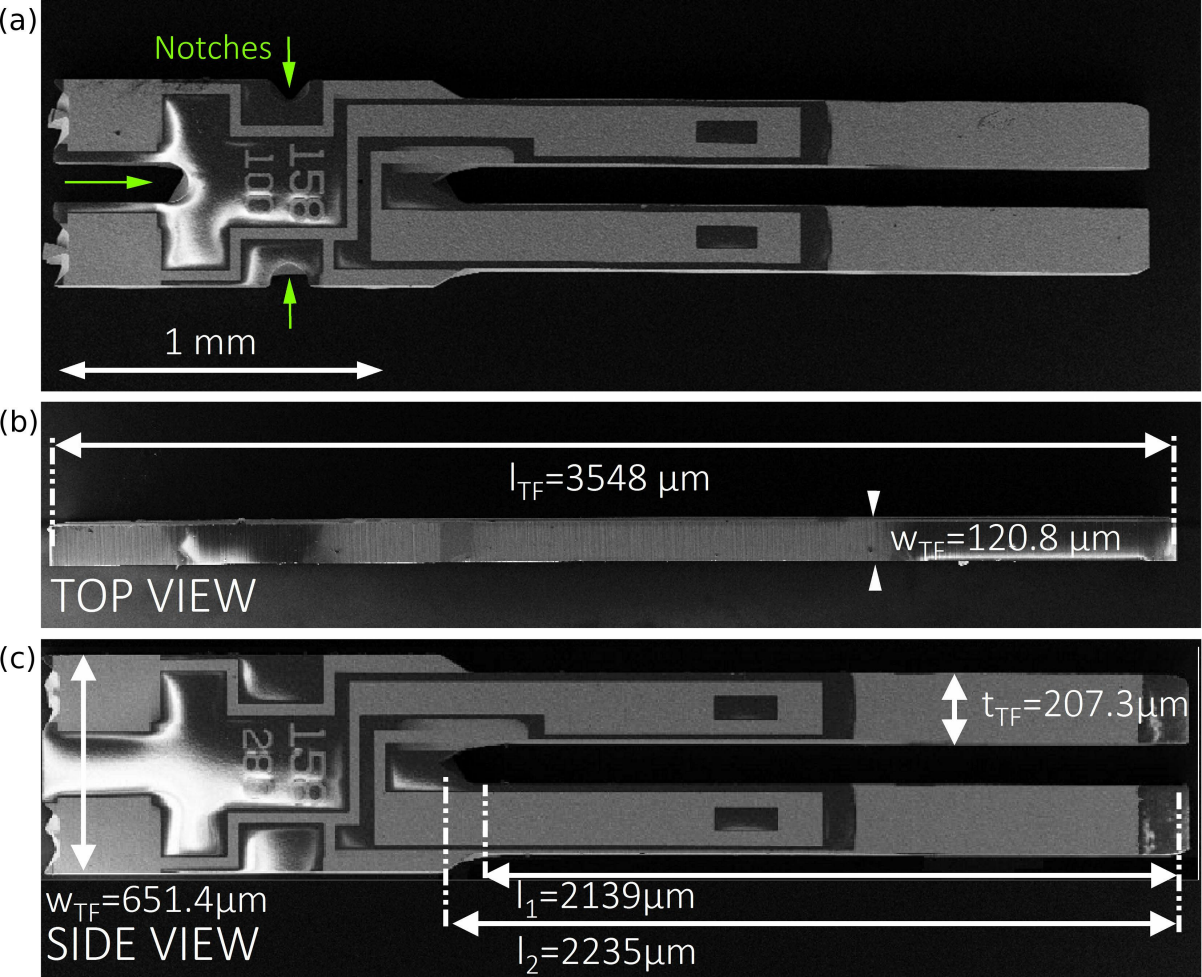
Determination of the geometric dimensions of a quarts tuning fork (Micro Crystal, type *DS*26 used for “qPlus”-force sensors from SEM images. (a) Sideview of tuning fork made from quartz with notches (cf. arrows) at the basis. (b) Topview of the tuning fork for measureing its width by the wafer thickness. (c) Sideview of an alternative geometric layout of DS26-type tuning fork without notches at the basis.

The quartz tuning fork, originally used as frequency normal in wrist watches constitutes the centerpiece of a force sensor in the “qPlus” design. Figure 1 shows micrographs from scanning electron microscopy (SEM) of a bare tuning fork (type DS26, Micro Crystal AG, Switzerland). These tuning forks are microfabricated from piezoelectric quartz, which is electrically contacted by gold electrodes placed onto the quartz substrate. The dimensions of the tuning fork can be easily measured by using SEM images as illustrated in Figure 1a and Figure 1b. The tuning fork has an overall length of *l*_TF_ = 3548 μm and a height of *h*_TF_ = 651.4 μm at the widest point while the substrate has a thickness giving the tuning forks width of *w*_TF_ = 120.8 μm and a prong thickness of *t*_TF_ = 207.3 μm. Figure 1c was taken from a derivative of the same type of tuning fork which differs only by the absence of notches at the basis compared to the tuning fork in panels a) and b) (compare arrows in panel a)). At this point it should be noted that all experiments and simulations presented here were carried out for both types (with and without notches). However, no differences were found in the stiffness of the sensors of the two types and therefore only one set (without notches) is presented here. In the “qPlus” design of nc-AFM force sensors, one prong and the end of the basis are fixed onto a carrier (usually from Macor) with epoxy glue. This type of fixation breaks the original quadrupole symmetry, in which both prongs oscillate around a forceless point that is found within the quartz body between the prongs. A very sharp tip etched from metal wire is attached to the end of the free prong, again with epoxy glue.

Commonly, spring constants of *k*_qPlus_ = 1800–2000 N/m are used for the force transformation. These values are estimated from the geometric dimensions of the free prong of the tuning fork and the Young’s modulus of quartz by using the beam formula according to Equation 1 [16].

[1]
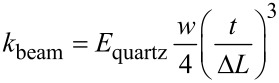


In this equation *w* and *t* are the width and thickness of the free prong, respectively and *E*_quartz_ is the Young’s modulus of quartz. The limitations for the validity of this formula are small deformations leading to only elastic stress/stain inside the uniform, rectangular cross section of the beam, which consists of isotropic material and is rigidly fixed at the end. These conditions are not necessarily fulfilled for a real tuning fork sensor. Since the tip wire is not necessarily placed at the very end of the prong, Δ*L* = *L* − *L*_0_ denotes the effective length of the free beam, i.e., the wire position *L* along the prong with respect to the beam origin *L*_0_. The comparison with Figure 1a shows that a certain ambiguity exists in the position of this beam origin *L*_0_. At the beam base the cross-section of the prong broadens before ending into the rigid basis. We here choose the point before the broadening as the zero point *L*_0_ as it is commonly done in the nc-AFM literature in order to avoid inaccuracies in later discussions. Inserting our measured values of Δ*L*_1_ = 2139 μm, *w* = 207.3 μm and *t* = 120.8 μm into Equation 1 together with the Young’s modulus of quartz of *E*_quartz_ = 78.7 GPa results in a stiffness of the free prong of *k*_qPlus_ = 1898 N/m. This is within the range of reported spring constant values *k*_qPlus_ = N/m [5] and *k*_qPlus_ = 2000 N/m [9], while the latter was calculated with a different Young’s modulus of *E*_quartz_ = 79.1 GPa to correct for the non-orthogonal crystallographic cut through the substrate of the tuning forks.

**Figure 2:**
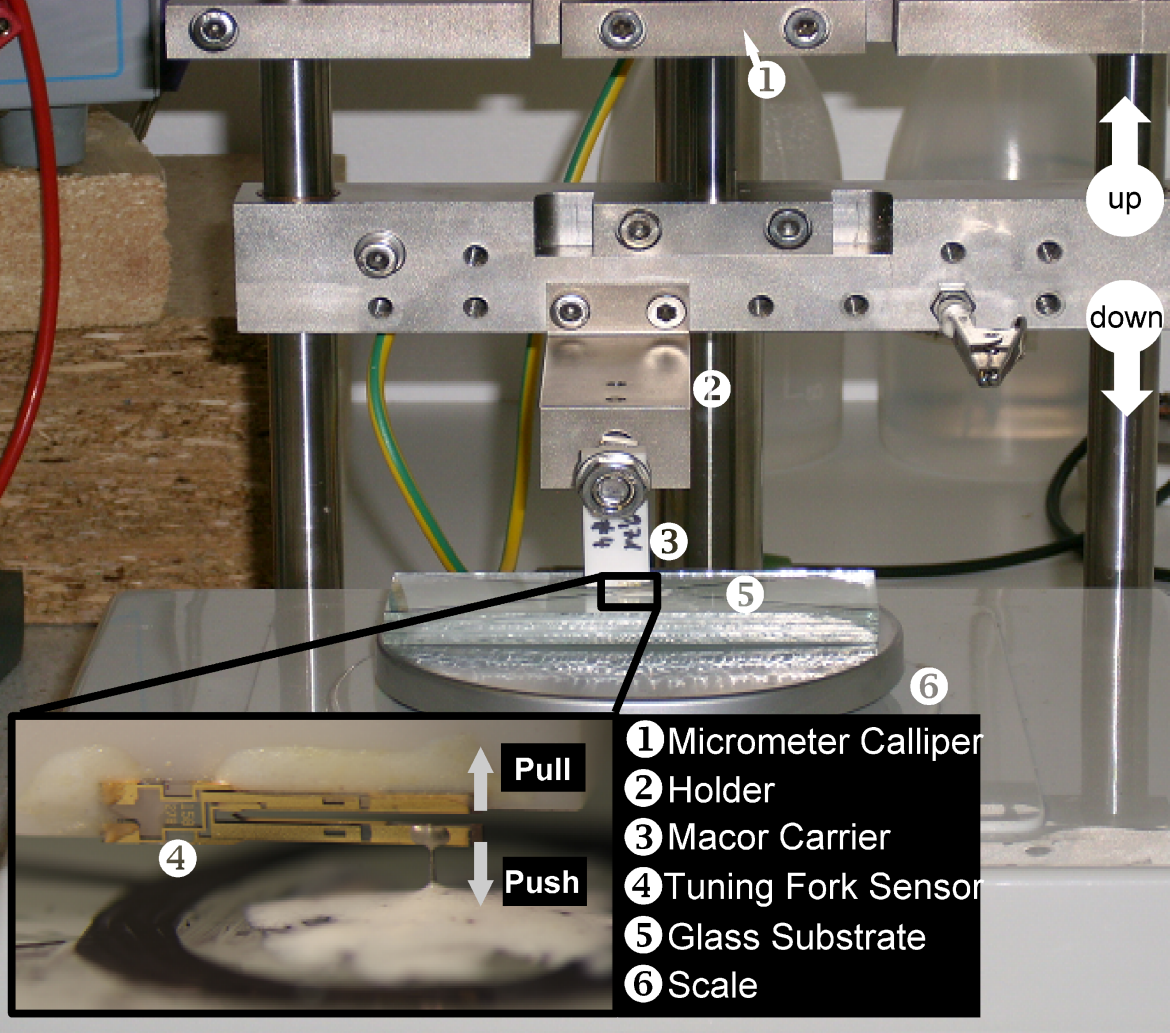
Photograph of the experimental setup. Not shown in the picture is the micrometer screw 1, which pushes or pulls the whole setup towards or from the scale. The Macor body 3, carrying the tuning fork sensor 4 is fixed to the holder 2. The inset shows a close-up of the tuning fork. The tuning fork is glued onto a Macor basis as in actual force sensors while the wire tip at the free prong is glued to a glass substrate. Latter transfers the force to the scale and delivers the mass for also pull the sensor away from the scale.

However, the underlying models of these calculations are barely in agreement with the actual geometry of real “qPlus” sensors, in which the force is applied through a metal wire glued onto the free prong. Therefore, the force application point is defined by the position of the glue point. Since these sensors are handmade it is obvious that the length Δ*L* cannot be regarded as constant for all sensors. The broadening of the beam towards the basis and the unknown Young’s modulus of the material limit the usage of the beam formula for the description of the tuning fork stiffness. Even influences of the glue, which is used to fix the tuning fork onto its holder, and the resulting spread in the individual stiffness of these sensors have recently been reported [17]. Possible methods to determine the stiffness are adding some mass to the prong and analyze the change of the dynamic oscillation [15] or static deflection [17–18] of the cantilever. Alternatively, the stiffness can be estimated from thermal excitation [19]. Here we employed a very simple and easily implementable method to measure the stiffness of the tuning fork sensors by only using a micrometer screw and a scale. The setup for such a measurement is shown in Figure 2.

In order to validate this measurement method we assembled a test sensor similar to the “qPlus” sensor setup. In the same way as in a “qPlus” sensor, a quartz tuning fork was glued onto a Macor body, and a tungsten wire with a diameter of *d*_W-wire_ = 50 μm was glued onto the free prong. This sensor is mounted onto a traverse, which can be lowered by a micrometer screw (Mitutoyo, type 110-164) with an accuracy of Δ*z* = 5 μm. Below the moveable traverse, a scale is placed (KERN & Sohn GmbH, type: KB 120-3) with a mass resolution of Δ*m* = 1 mg. The force applied to the scale is then calculated by multiplying the weight with the gravitation constant *g* = 9.81 m/s^2^ resulting in an accuracy of the force measurement of 9.81 μN. The stiffness of the sensor can now be measured by pushing the sensor onto the scale with the micrometer screw while simultaneously measuring the weight increase on the scale. By lowering the end of the wire into a fresh droplet of Torr Seal epoxy glue, it can be mechanically fixed onto a glass substrate resting on the scale (cf. Figure 2, inset). After the glue is cured out at room temperature, the stiffness can be measured in both directions, pushing (increasing mass on scale) or pulling (decreasing mass on scale). Please note that during a pull-experiment under the present conditions the relative elongation of the tungsten wire remains lower than 0.1% and is therefore neglected in the further analysis. A reference experiment was performed with a bare Macor carrier (without tuning fork) to measure the stiffness of the experimental setup *k*_setup_ (mainly the compliance of the scale), which was in our case *k*_setup_ = 5952 N/m. The stiffness of the tuning fork can then be evaluated by Equation 2 representing a series of both stiffnesses.

[2]
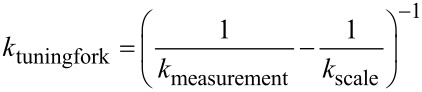


**Figure 3:**
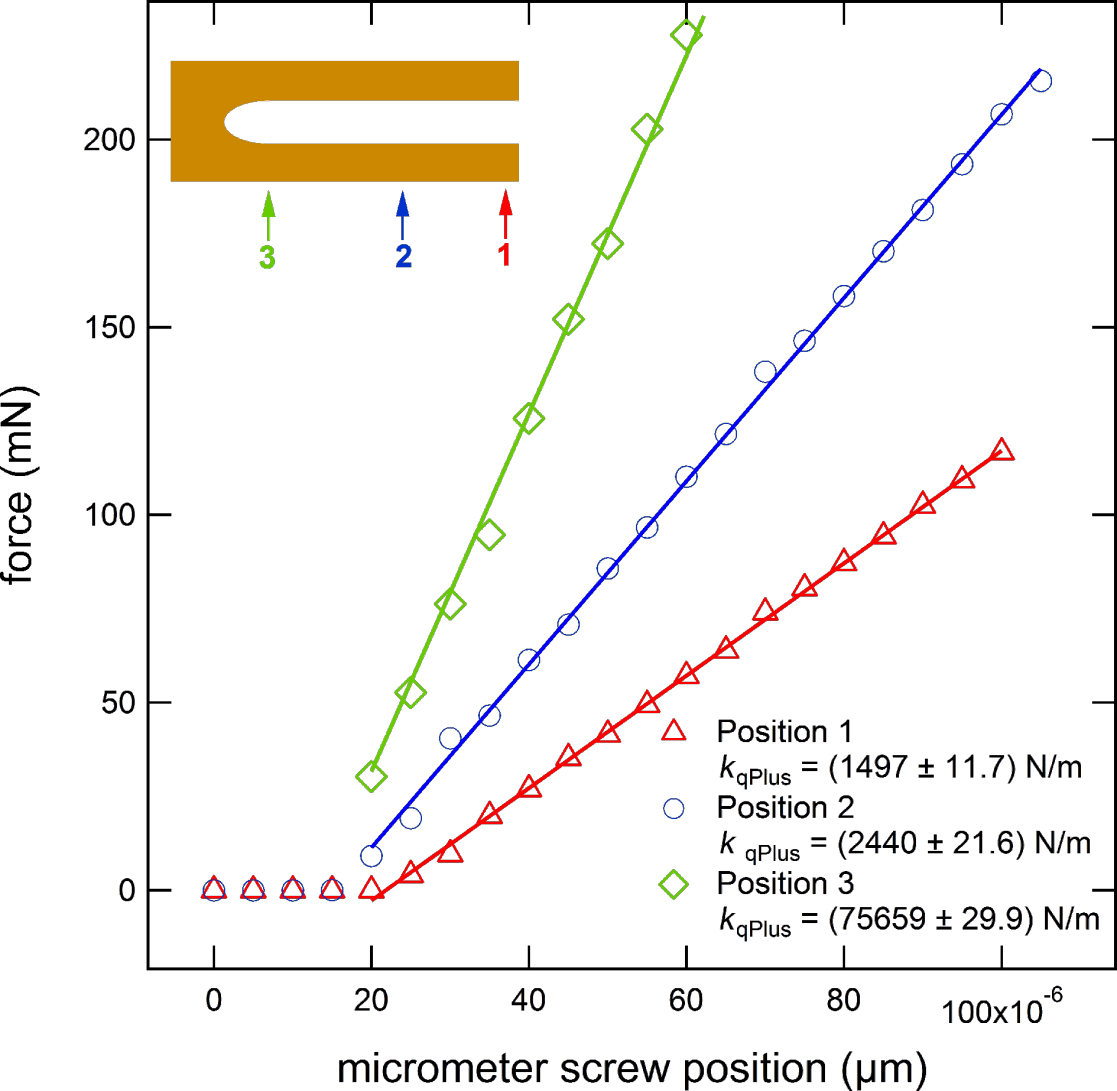
Diagram of a “push” experiment to measure the stiffness of the free prong of a “qPlus” sensor by the slope of the fit to the data points with an error of approx. 1%. The deflection of the prong starts at position 20 μm of the micrometer screw. The spring constant had been calculated in the range of increasing forces. The effective stiffness increases for decreasing effective prong length, i.e., for tip positions located further to the beginning of the prong. The stiffness was calculated from the slope.

With the setup described above, the stiffness of the bare tuning fork was measured as a function of the position of the force application point, i.e., the tip wire. The diagram in Figure 3 shows data points recorded by pushing at different positions along the tuning fork prong. The deflection of the tuning fork rises with increasing the position of micrometer screw, starting from the point of contact at a position of 20 μm. The stiffness of the sensor can be evaluated by fitting these data by the solid lines within an error of less than 1%. The position was determined from photographs taken through a stereo microscope during the pushing experiment. The result of the position dependence is then compared with the values predicted by the beam formula (Equation 1) while using the effective beam length Δ*L* = *L* − *L*_0_ with respect to the force application point *L*. Table 1 lists the measured stiffness values as well as the values calculated from the beam formula. While for long prongs (large Δ*L* values), the measurement seems to be roughly within the range of the calculation, for shorter prongs (small Δ*L*) a drastic discrepancy between the measured stiffness and the calculated value is found (up to a factor of 5 or larger, cf. last column of Table 1).

**Table 1:** Comparison of the measurement of the stiffness with the calculation using the beam formula for the identical position at the prong.

position (μm)	measured spring constant (“push” experiment) (N/m)	calculated spring constant (beam formula) (N/m)	measured value/calculated value

408	65427	357386	0.18
604	33784	120717	0.27
1062	16150	17799	0.91
1085	6315	20576	0.31
1630	3088	4986	0.62
1653	4135	4719	0.88
1994	2000	2690	0.74
2052	2892	2460	1.18

In fact, a deviation between the experimental tuning fork stiffness and the beam formula is not unexpected. Previous simulations suggest that the zero point has to be chosen differently as it is commonly done when using the beam formula [20]. These findings motivated our detailed analysis of the mechanical tuning fork properties by FEM using the software Comsol Multiphysics (V 4.1a). In addition to the measurement of “custom-made qPlus” sensors, we also measured the spring constant of “qPlus” sensors from Omicron NanoScience GmbH, Taunusstein. The result is that even these sensors show a significantly high spread of *k*_qPlus_ = 1480–1708 N/m, which demonstrates the need to calibrate each individual sensor that is used for quantitative nc-AFM force spectroscopy measurements.

### FEM simulations

**Figure 4:**
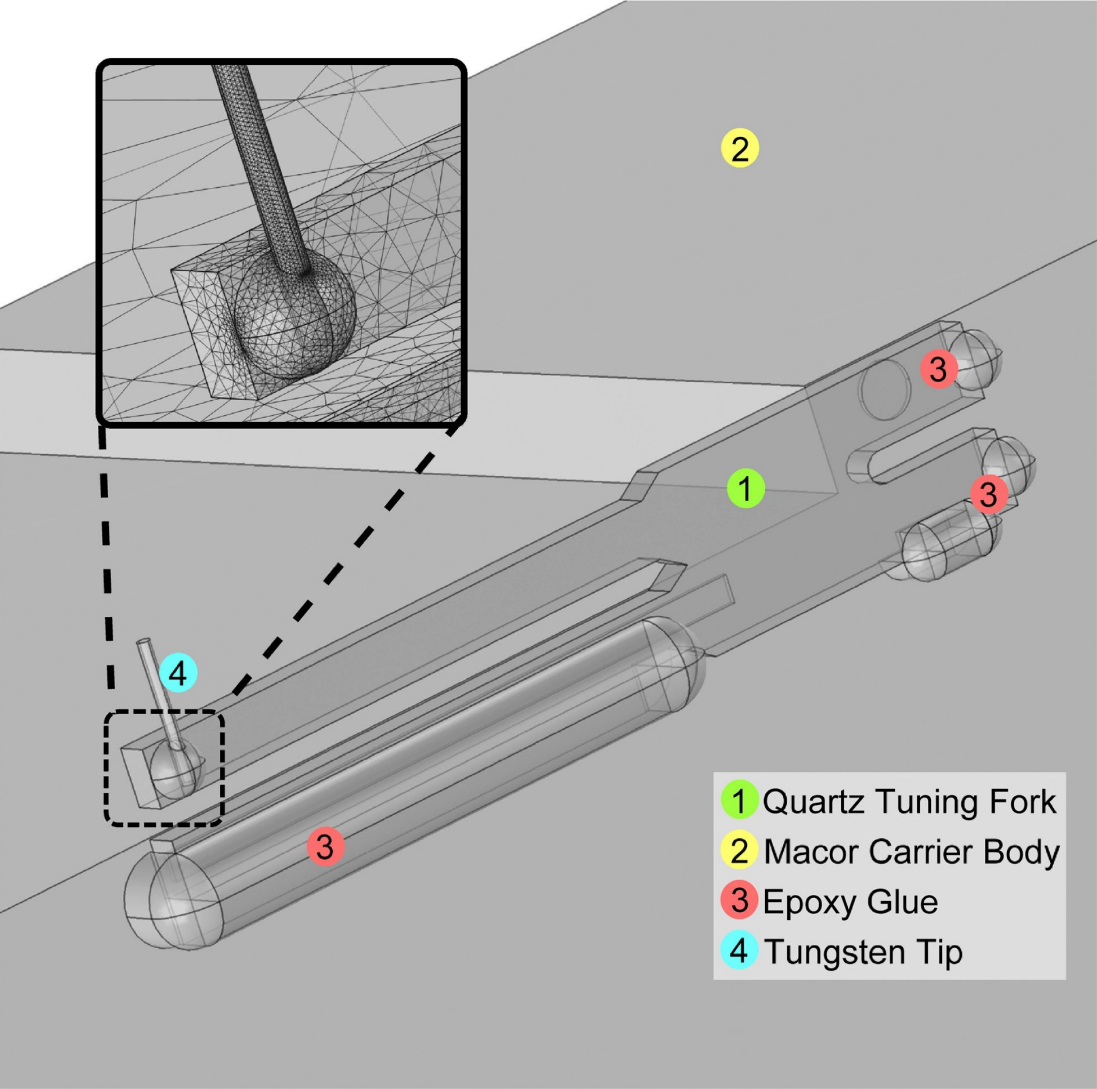
Image of the geometric model reflecting the geometry of an actual “qPlus” sensor. The model includes a tip (4) attached to the free prong with a droplet of epoxy glue, as well as the epoxy glue (3) at the rim and behind the tuning fork (1) fixing it to the Macor carrier (2). The sophisticated geometry is meshed with a tetrahedral elements (cf. inset) to better account for the transition between the individual geometry elements. The material properties were taken from literature, as for the Young’s modulus of the epoxy glue, tensile experiments were carried out to determine a realistic value for the crucial connection of the force application point between the metal tip and the prong.

Special care was taken to make the geometric model of the “qPlus” sensor in the FEM software as realistic as possible, including gluing points as well as a metal tip. As for the tuning fork, an isotropic Young’s modulus of *E*_quartz_ = 78.9 GPa was used. To obtain a realistic value of the Young’s modulus of the glue for the FEM simulations, three samples made from “Torr Seal” were tested in a tensile test sample geometry accordant to DIN EN ISO 527 in a tensile test. Two of the Torr Seal samples were cured at a temperature of 100 °C resulting in *E*_TorrSeal_ = 6500 GPa and 6000 GPa, respectively. The third sample was cured at room temperature (RT) resulting in a Young’s modulus *E*_TorrSeal,RT_ = 4000 GPa. As our custom-build “qPlus” sensors are cured out in an oven, the value of *E*_TorrSeal_ = 6000 GPa was used in our FEM simulations for the epoxy glue. The geometry of the simulated model is depicted in Figure 4 in more detail. The sophisticated geometry of different sub-geometries, is meshed by tetrahedral elements, which allow a very fine mesh at the boundary lines as well as the boundary areas between the sub-geometries (in particular at the force application point from the wire through the glue droplet into the free prong).

**Figure 5:**
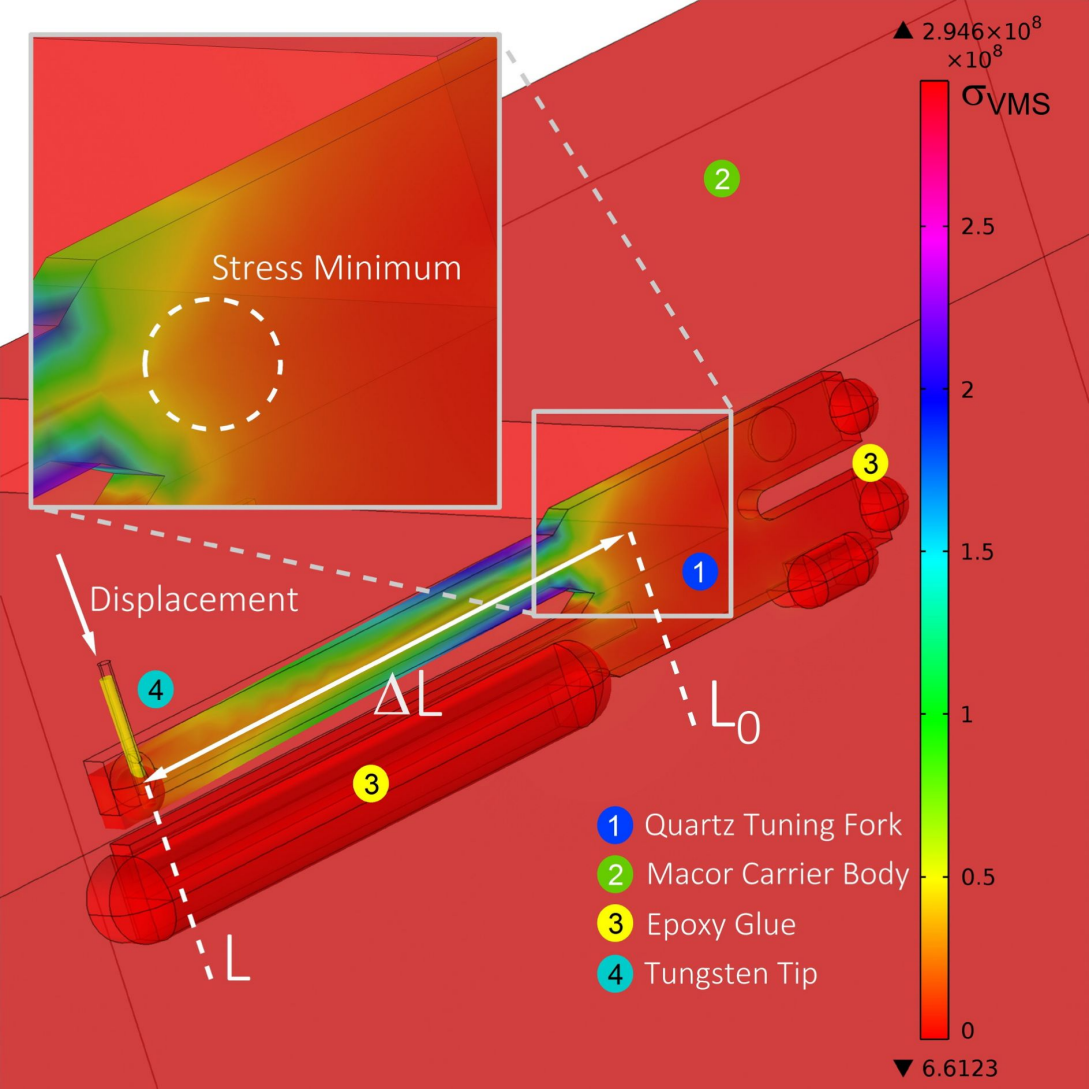
FEM simulation of von Mises stress. Analysis of the stress caused by the bending of the free prong. In contrast to the model for the beam formula, in which a cantilever is fixed at one end, the stress in the quartz tuning fork reaches beyond the end of the prong far into the basis of the tuning fork. The origin of the minimal Van Mises stress is indicated by the dashed circle (see inset).

In the next step a force was applied through the vertical axis of the wire and the displacement of the free prong was analyzed. Interestingly, a closer look at the stress distribution reveals that the stress is reaching several hundred microns into the basis of the tuning fork. Figure 5 shows the stress distribution within the tuning fork caused by a loading force of *F*_load_ = 1–100 mN, which results in a displacement of the very end of the free prong of *x*_end_ = 50 μm. Since the tip was attached to the side of the tuning fork, as it is also the case in commercial “qPlus” sensors, the different stress contributions of torsional and normal stress are color coded as the comparative von Mises stress (σ_VMSmin_). The color code represents stress values from σ_VMSmin_ = 0 N/m^2^ (red) to maximum values of σ_VMSmax_ = 2.5·10^8^ N/m^2^ (violet). The area of onset of stress within the basis is marked by the dashed circle.

**Figure 6:**
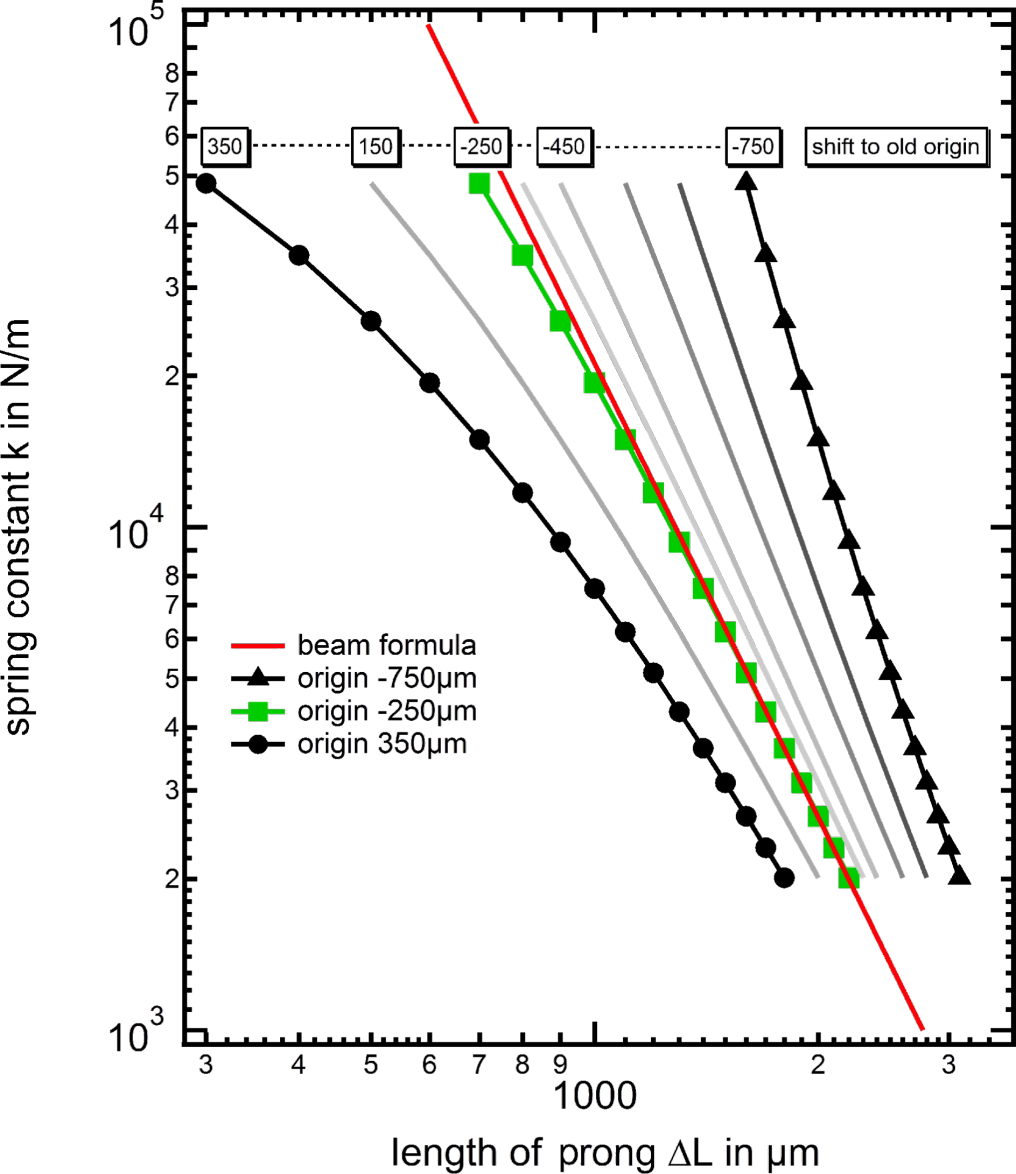
Diagram showing the results of the FEM simulation as a function of the shift of the origin. While for too large or too small chosen positions of the origin the curves show a non linear behavior, the a Δ*L*^−3^ behavior can be identified in the transition regime for a new effective origin position of approx. *L*_0_ = −250 μm.

This finding suggests that the zero point *L*_0_, as origin for the length of the cantilever, has to be adjusted when calculating the stiffness of a tuning fork by using the simple beam formula. To demonstrate this effect we first plot the stiffness of the tuning fork in Figure 6 using the zero point at the end of the narrow beam, i.e., *L*_0_ = 0 as a reference curve. The logarithmic plot shows that the spring constant versus beam length curve (gray curves) does not follow a certain power law, e.g., Δ*L*^−3^ as expected from Equation 1. For direct comparison we also plotted the results from the beam formula of Equation 1 as a red solid line. Motivated by the non-negligible stress reaching into the tuning fork basis, the results of the FEM simulation are plotted for different beam origins *L*_0_ + Δ*L*_0_ reaching into the base of the tuning fork. The resulting set of curves is plotted in the diagram of Figure 6, where the new effective origin *L*_0_ was adjusted to a range from 350 μm to −750 μm. Here, in the transition regime between the two extreme *L*_0_ positions, a linear behavior can be identified at an effective origin of *L*_0_ = −250 μm, which is located “inside” the basis of the tuning fork with respect to the initial origin at *L*_0_ = 0. For the new origin *L*_0_ = −250 μm we find quantitative agreement between simulations and beam formula for larger tip wire positions Δ*L* > 1500 μm, which is the case in conventional “qPlus” sensors but also indicates that additional care has to be taken when working with shorter prongs. Only if the tip wire is closer to the basis, some deviations occur, in which the beam formula is systematically overestimating the stiffness. Therefore we conclude that the beam formula can still be used to estimate the tuning fork prong spring constant, if the beam origin is set to the new effective position *L*_0_ = −250 μm (for the tuning forks used here) and if the tip wire position is more than 1500 μm away from the origin.

**Figure 7:**
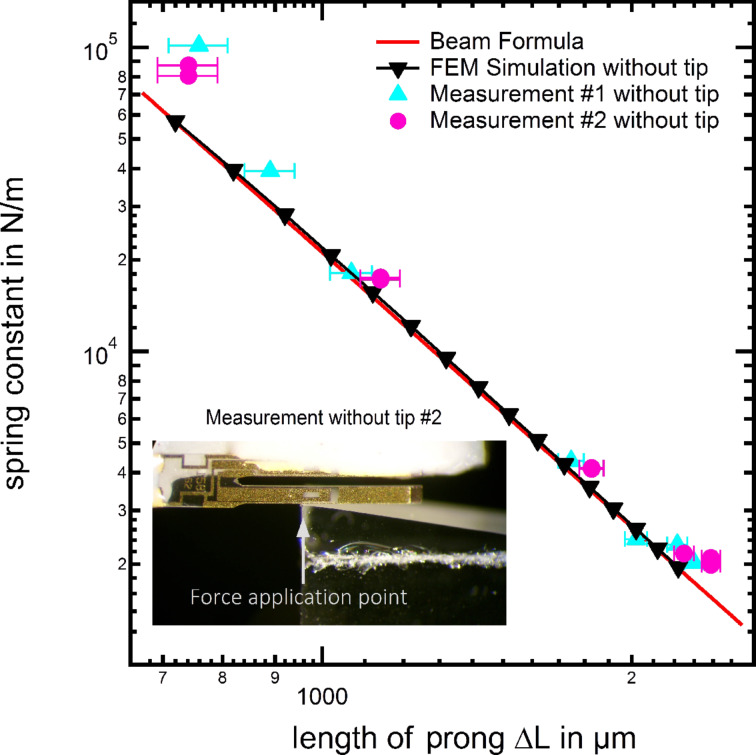
Comparison between the beam formula, experimental measurements and FEM simulation with the force directly applied to the tuning fork prong. The prior introduced origin shift has already been applied to the beam formula resulting in a higher compliance of the plot here.

In the following, the still existing deviation between the FEM results and the beam formula, is subject to further investigations. Therefore we simplify our experimental and FEM setup. To eliminate a possible influence caused by the tip, we carried out two separate measurement series to determine the spring constant directly by applying a force onto the top of a tuning fork sensor prong, together with an analogue FEM simulation. The experimental setup and the corresponding results are displayed by the graph and the photograph in Figure 7. The graph shows a high agreement between the FEM and experimental results with the beam formula, clearly identifying the tip as source for the discrepancy discovered in Table 1 and Figure 6. One reasonable explanation for the occurring discrepancy is the additional torsion induced into the prong by the wire attached at the side of the free prong.

**Figure 8:**
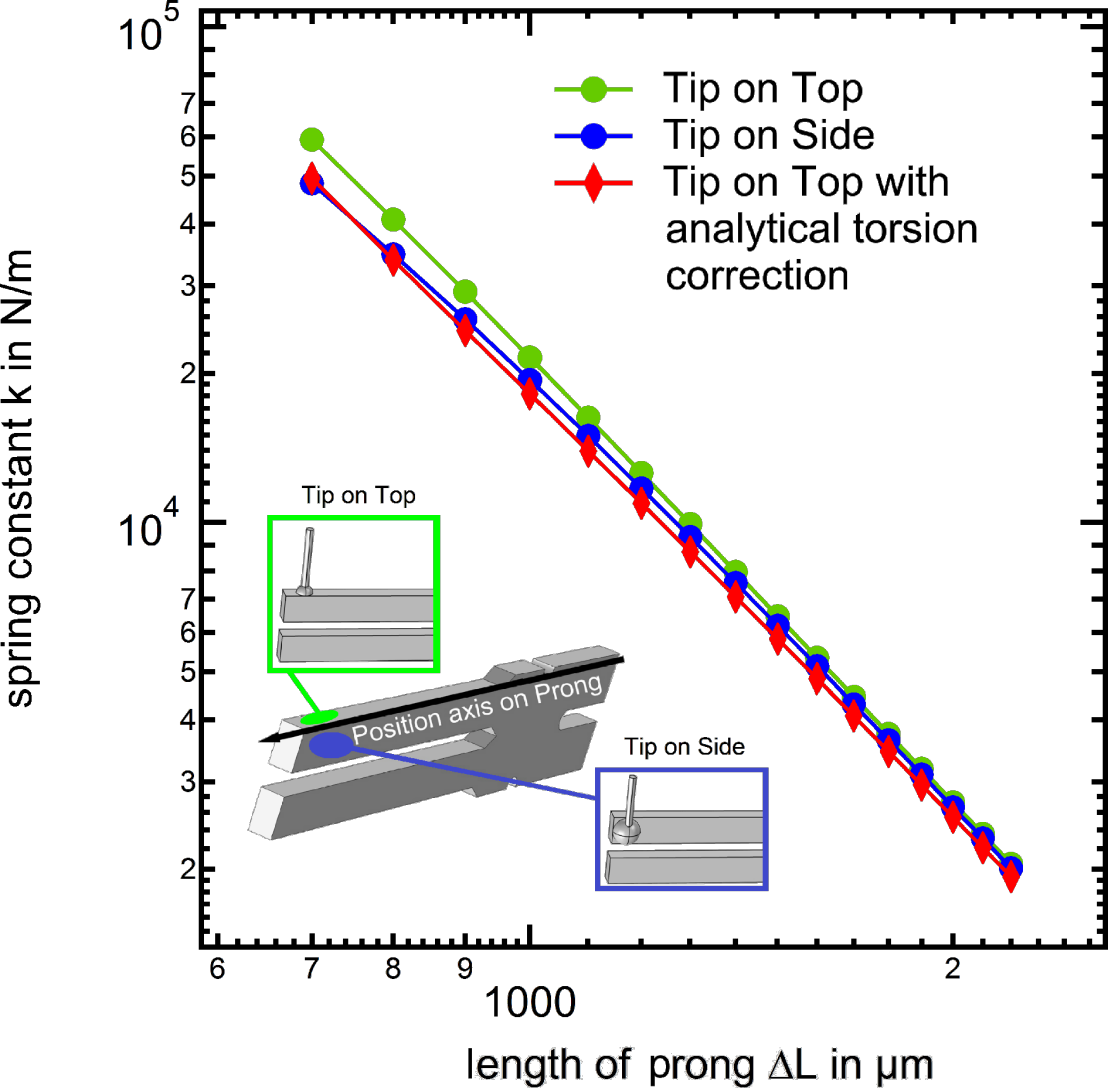
Comparison between tip on side (TOS) and tip on top (TOT) configurations as possible origin of the deviation between FEM Simulation and Experiment. The deviation was found to be larger in case of TOT than the contribution of torsion in the TOS configuration.

Subsequently we investigate the influence of torsional motion of the tuning fork prong, which may also play an important role. While the beam formula only considers normal forces applied orthogonal to the axis of the prong, in the “qPlus” sensor configuration, the wire-tip is attached at the side causing a torque around the axis of the beam in addition to the bending of the prong. To evaluate the influence of the torsion, the simulation was repeated with the tip positioned at the center of the prong (indeed some experimentalists attach the wire-tip on the face side of the free prong to avoid torsion during the AFM-experiments). In our FEM simulations the position was chosen with the tip on the top of the prong (TOT), allowing us to vary the position of the force application point, for direct comparison to the results from the tip on side (TOS) configuration, which was discussed so far. Figure 8 shows the result of the FEM simulation in the two configurations, TOS (blue) and TOT (green). While for positions at large beam lengths, the deviation between the two configurations is negligible, in the regime of positions of short beam length values, a deviation can be noticed. The contribution caused by the torsion can be calculated analytically by the following relation [21]:

[3]
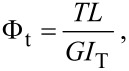


in which *T* is the torsional momentum, Φ_t_ is the angle of twist in radians, *L* the length at which the force is applied, *G* the shear momentum and *I*_T_ the second momentum of area of the prong. To calculate the exact influence of the torsion to the overall spring constant, the tuning fork has to be seen as a system of two springs (deflection and torsion) connected in series. The red curve in the diagram shows the result from the simulated TOT-configuration where the effect of the torsion is corrected with the above equation. The torsion corrected curve coincides well with the curve simulated for the TOS configuration of the “qPlus” sensor. These results also demonstrate that torsion has a negligible influence at the free end of the prong, since the torsion spring constant is decreasing linearly whereas the deflection spring constant decreases with Δ*L*^−3^. Only if the tip is mounted closer to the origin of the tuning fork body, the torsion has an increasing influence on the overall spring constant. This influence results in a smaller increase of the overall spring constant as the torsion spring constant is not increasing as fast as the deflection spring constant. This effect is obvious in the area of smaller Δ*L*-values, in which the TOS curve shows a recognizably lower spring constant, than the TOT-curve.

**Figure 9:**
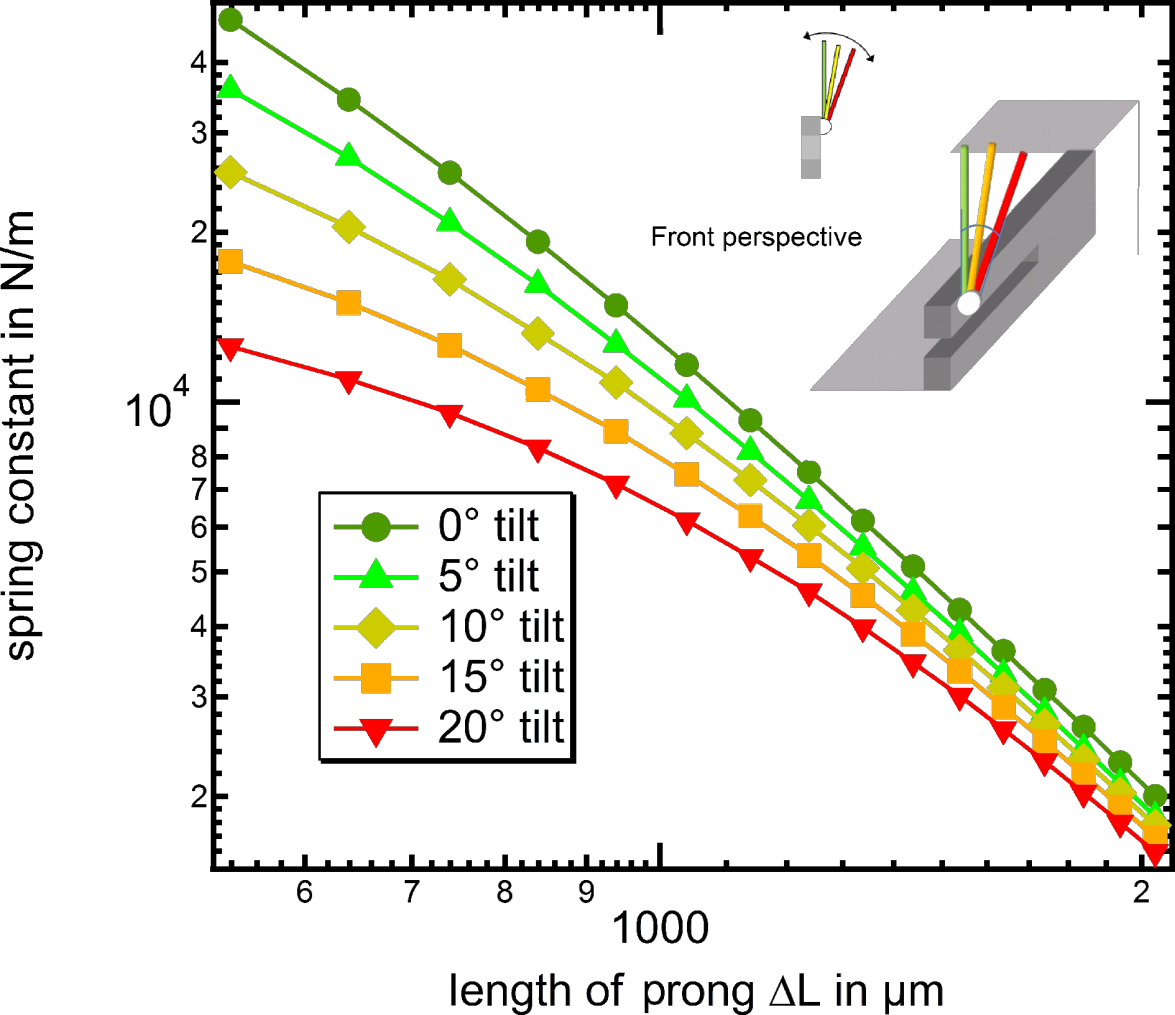
FEM simulation result displaying the influence of a tilted tungsten wire on the resulting spring constant versus a non tilted wire. It is obvious that a strongly tilted tip causes an increasing influence of torsion. Thus during the assembly one should also focus on the angle between tuning fork and wire trying to keep it as small as possible.

Before we proceed by finally comparing the results of the FEM calculations and the modified beam formula with the experimental spring constants, we consider one further important issue related to the hand-made “qPlus” sensor fabrication. Since the wire is glued on the prong, very often a small tilt of the wire long axis with respect to the prong surface normal cannot be excluded. Unfortunately, the torsion caused by the non central fixation of the tungsten wire is increasing, when the wire is not perpendicular mounted to the tuning fork. Therefore we conducted further FEM simulations considering a possible wire axis tilt, with the results shown in Figure 9. This figure demonstrates clearly that even a small misalignment of the wire axis can lead to large deviations of the effective spring constant, in particular for wire fixation points close to the tuning fork base.

**Figure 10:**
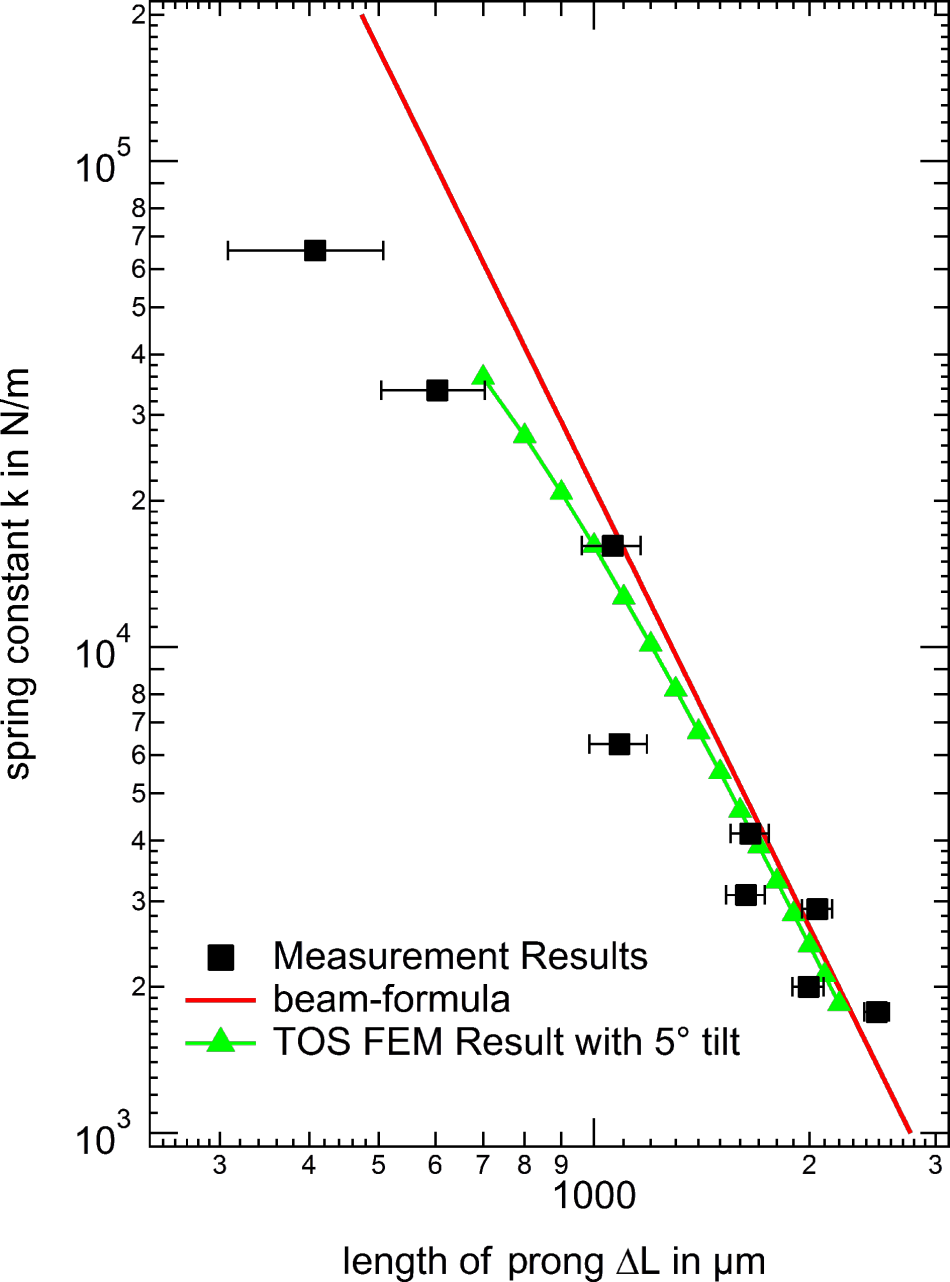
Comparison of the spring constants from experiment with FEM simulations and calculations using the beam formula (with the new origin *L*_0_ = −250 μm for both). The experimental values still shows a deviation from the simulations and calculation respectively. This is possibly caused by some tilt of the sensor towards the force application axis where small angles cannot be completely avoided in the fabrication procedure.

As the final step, Figure 10 now displays the comparison between the experimental results (black square markers), the FEM simulations including a small 5° tilt (green triangles) and the modified beam formula (red line). First we note that the experimental spring constant results show a considerable spread, in which almost identical tip positions may still result in differences of a factor of three in the most extreme cases, while differences of 50% are typically found. This spread in the individual spring constants is most likely due to tip axis misalignment during the “qPlus” fabrication. Even when carefully assembling the tuning fork sensors under optical microscope inspection misalignment angles of up to 10° are common. In fact the spread in spring constants agrees with the range of misalignment angles considered in Figure 9. Despite this scatter in the individual data we find overall that there is a decent agreement between the measured spring constant values and the FEM results with a 5° tilt included, which is a realistic average value for careful manual tip fixation procedures. Furthermore, we can now directly compare how well the origin shifted beam formula agrees with FEM data and experimental values. Again, in the regime of large Δ*L* values (Δ*L* > 1500 μm) the agreement between experiment and simulations/beam formula is acceptable, if the shifted origin method is applied. Please note that the scatter between the individual experimental data points is larger than the difference between beam formula and FEM data with 5° tilt angle.

From this section we conclude that using the conventional beam formula for the calculation of the spring constants of tuning forks results in a dramatic overestimation of the beam compliance. However, the origin shifted beam formula can be used to estimate the “qPlus” spring constant for Δ*L* > 1500 μm. Still in this case a typical error of about 50% remains, which is mainly due to angular misalignment effects during the tip wire fixation to the free prong. For more precise spring constant determination, as required for quantitative force spectroscopy experiments, individual calibration of the used tuning fork sensors after the nc-AFM experiment is mandatory.

## Conclusion

A simple method for measuring the spring constant of tuning fork sensors using a micrometer screw and a scale is presented. The experimental results are compared to the beam formula and FEM-simulations revealing the limits of the commonly used models for the determination of “qPlus” sensor stiffness. The combination of finite element method simulation with experimental measurements allows a comprehensive understanding of the spring constant behavior alongside the whole length of the free prong. This knowledge finally opens the opportunity to adapt the beam formula by shifting the origin of the beam formula and thus making it a reliable tool for the spring constant determination in the area around the last millimeter of the prong. Since the beam formula is calibrated by the present study, it can be used for the determination of spring constants of “qPlus” sensors by measuring the effective length between the force application point at the gluing droplet attaching the wire to the prong and the shifted coordinate for the zero point of Δ*x*_0_ = −250 μm into the basis. This length can either be measured from SEM images of tuning fork sensors or even simpler by microscopic photograph. However, the present study reveals that the stiffness of real sensors can differ from the simulations due to deviations between the real tuning fork tip alignment and the ideal FEM model geometry. Whenever a more precise value of the static spring constant is required, due to the significantly large spread of the experimental results, the presented method to measure the stiffness directly can be applied to the sensor after the AFM spectroscopy experiment.
